# Measuring Epidemiologic Effects of Enterococcal Bacteremia and Outcomes From a Nationwide Inpatient Sample Database

**DOI:** 10.7759/cureus.27516

**Published:** 2022-07-31

**Authors:** Pramil Cheriyath, Ankita Prasad, Premalkumar Patel, Varun Vankeshwaram, Sheilabi Seeburun, Kajal Ghodasara, Sandeep Pavuluri

**Affiliations:** 1 Internal Medicine, Hackensack Meridian Ocean University Medical Center, Brick, USA; 2 Infectious Disease, Mount Sinai Medical Center, Miami Beach, USA; 3 Medicine, Zaporozhye State Medical University, Zaporizhzhia, UKR; 4 Internal Medicine, Dr AG (Abdool Gaffoor) Jeetoo Hospital, Port Louis, MUS

**Keywords:** healthcare cost, bacteremia, length of stay, cardiac, renal, national inpatient sample database, morbidity, mortality, enterococcus

## Abstract

Introduction

*Enterococcus *is a gram-positive, non-sporing, facultative anaerobe. It is a common cause of nosocomial infections in the United States. Enterococcal bacteremia is primarily a nosocomial infection in the medical intensive care unit (ICU), with a preference for elderly patients with multiple comorbidities.

Material and methods

This is a retrospective cohort study using the publicly accessible National (Nationwide) Inpatient Sample (NIS) database from October 2015 to December 2017. We examined data from 75,430 patients aged 18 years and older in the NIS who developed enterococcal bacteremia, as identified from the ICD-10 CM codes (B95), to discuss the epidemiologic effects and outcomes of enterococcal bacteremia. Patients were classified based on demographics, and comorbidities were identified. Three primary outcomes were studied: in-hospital mortality, length of stay, and healthcare cost. The secondary outcome was identifying any comorbidities associated with enterococcal bacteremia. Length of stay was defined as days from admission to discharge or death. Healthcare costs were estimated from the hospital perspective from hospital-level ratios of costs-to-charges. SAS 9.4 (2013; SAS Institute Inc., Cary, North Carolina, United States) was used for univariate and multivariate analyses. For data analysis, mortality was modeled using logistic regression. Length of stay and costs were modeled using linear regression, controlling for patient and hospital characteristics. Statistical analyses were performed using SAS. Statistical significance was defined as P<0.05.

Results

A total of 75,430 patients with enterococcal bacteremia were included in the study. Of this, 44,270 were males and 31,160 females. A total of 50,270 (68.67%) were Caucasians, 11,210 (15.31%) were African Americans, 6,445 (8.80%) were Hispanic and 2,025 (2.77%) were native Americans. Important comorbidities were congestive heart failure (25.91%), valvular disease (8.08%), neurological complications (11.87%), diabetes mellitus with complications (18.89%), renal failure (28.52%), and obesity (11.61%). In-hospital mortality was 11.07%, length of stay was 13.8 days, and a healthcare cost of 41,232.6 USD.

Conclusions

Enterococcal bacteremia is a nosocomial infection with a preference for the elderly with renal failure, cardiac failure, cardiac valvular diseases, stroke, obesity, and diabetes with complications. Further studies are needed to see whether the mortality caused by enterococcal bacteremia is attributable to comorbidities or to the bacteremia. It is associated with a more extended hospital stay and higher healthcare expenditure. Implementing contact precautions to contain the spread of methicillin-resistant *Staphylococcus aureus* (MRSA) and vancomycin-resistant *Enterococcus*(VRE) has also checked the spread of enterococci. Further prospective studies can be planned using chart-based data.

## Introduction

*Enterococcus *is a gram-positive, non-sporing, facultative anaerobe and a common commensal in the human gastrointestinal (GI) tract. *Enterococcus* *faecalis* and *Enterococcus* *faecium* are the most frequent causes of invasive infections. They are the first organisms of the ESKAPE group (*Enterococcus, Staphylococcus aureus, Klebsiella pneumoniae, Acinetobacter baumannii, Pseudomonas aeruginosa, Enterobacter*) that the World Health Organization (WHO) considers a vital source of healthcare infection [1]. *Enterococcus* is the third most common cause of nosocomial diseases in the United States, following *Staphylococcus *and coliforms* *[2]. 

Enterococcal bacteremia is primarily a nosocomial infection in the ICU, mainly in the elderly with multiple comorbidities. It is associated with a more extended ICU stay and higher mortality [3]. The increased incidence of enterococcal bacteremia in the elderly may be related to monitoring with invasive vascular devices, indwelling urinary catheters, and skin breakdown at pressure sites. Enterococcal bacteremia is frequently associated with bacterial endocarditis, urinary tract infection (UTI), meningitis, and spontaneous bacterial peritonitis. It* *can cause up to 30% of all endocarditis [4]. The primary sources of enterococcal bacteremia are the urinary tract following catheterization, soft tissue infections, and intra-abdominal infections following surgery, in which case the disease tends to be polymicrobial [5].

*Enterococcus* is resistant to a wide range of temperatures, pHs, and salt concentrations. Itsvirulence comes from its structure, its ability for biofilm formation, and an inherently high degree of antibiotic resistance. *Enterococcus* surface components include the polysaccharide capsule, pili, aggregation substance, and adhesins, which cause attachment to host tissues and form colonies; the biofilm then causes bacterial adhesion and persistent infections.

* *In our study, we want to find the epidemiologic effects of enterococcal bacteremia on mortality, length of stay, cost of hospitalization, and any comorbidities that have an increased association with enterococcal bacteremia.

## Materials and methods

This is a retrospective cohort study done to measure the epidemiological effects of enterococcal bacteremia using the publicly accessible National (Nationwide) Inpatient Sample (NIS) database from October 2015 to December 2017. The NIS database was developed through a Federal-State-Industry partnership sponsored by the Agency for Healthcare Research and Quality (AHRQ). Healthcare Cost and Utilization Project (HCUP) data are used in national, state, and community decision-making.

We examined 75,430 patients aged 18 years and older in the 2015-2017 NIS database who developed enterococcal bacteremia as identified by the International classification of diseases (ICD)-10 CM codes (B95). Enterococcal bacteremia is defined as *Enterococcus* isolated from one or more blood cultures. Patients were classified based on sex, race, geographical region (northeast, midwest, south, and west), income quartile by zip code (0-25th, 26-50th, 51-75th, 76-100th), insurance type (Medicare, Medicaid, private, or any other), hospital ownership/control (rural, urban nonteaching, and urban teaching), hospital bed size (small, medium, or large), and final disposition (whether discharged to home, home health care, or left against medical advice (AMA))

The study’s objective is to discuss the outcomes of enterococcal bacteremia using ICD-10 CM codes (B95) from the 2015-2017 NIS database. Three outcomes were studied: in-hospital mortality, length of stay, and hospital costs. Length of stay was defined as days from admission to discharge or death. Costs were estimated from the hospital perspective from hospital-level ratios of costs-to-charges. All charges were adjusted to 2018 US dollars using the medical care component of the consumer price index. 

Statistical analyses were designed to determine whether there was a significant association between the comorbidities and bacteremia. Mortality was modeled using logistic regression. Length of stay and costs were modeled using linear regression, controlling for patient, and hospital characteristics. Statistical analyses were performed using SAS 9.4 (2013; SAS Institute Inc., Cary, North Carolina, United States). Statistical significance was defined as P<0.0001.

## Results

There were 75,430 patients included in the study who developed enterococcal bacteremia during the inpatient stay. In this cohort, 44,270 (58.69%) were males and 31,160 (41.31%) females. Of the patients, 50,270 (68.67%) were Caucasians, 11,210 (15.31%) were African Americans, 6,445 (8.80%) were Hispanic, and 2,025 (2.77%) were native Americans (Table 1).

**Table 1:** Patient demographics

Demographics	Number of Patients
Male	44,270 (58.69%)
Female	31,160 (41.31%)
Caucasians	50,270 (68.67%)
African Americans	11,210 (15.31%)
Native Americans	2,025 (2.27%)
Hispanics	6,445 (8.80%)

Patients were further identified based on geographical region (northeast, midwest, south, and west), income quartile by zip code (0-25th, 26-50th, 51-75th, 76-100th), insurance type (Medicare, Medicaid, private, or any other), hospital ownership/control (rural, urban nonteaching, and urban teaching), hospital bed size (small, medium, or large), and final disposition (discharged to home, home health care, or left against medical advice (AMA)) (Table 2).

**Table 2:** Classification of patients on the basis of location, income quartile, insurance type, hospital type, size of hospital bed, and final patient disposition

Classification	Enterococcal bacteremia	p=Value
Geographical region		
Northeast	13,950 (18.5 %)	<0.0001
Midwest	16,975 (22.5 % )	<0.0001
South	28,110 (37.2 %)	<0.0001
West	16,430 (21.8 %)	<0.0001
Income quartile by zip code		
0–25^th^	21,715 ( 29.3 %)	<0.0001
26–50^th^	19,870 (26.8 %)	<0.0001
51–75^th^	17,315 (23.4 %)	<0.0001
76–100^th^	15,190 (20.5 %)	<0.0001
Insurance type		
Medicare	51,475 (68.2 %)	<0.0001
Medicaid	9,105 (12.1 %)	<0.0001
Private	12,020 (15.9 5 )	<0.0001
Other	1,525 (2.0 5)	<0.0001
Hospital ownership/control		
Rural	5,965 (7.9 %)	<0.0001
Urban nonteaching	17,005 (22.5 %)	<0.0001
Urban teaching	52,495 (69.6 %)	<0.0001
Size of hospital bed		
Small	13,410 (17.8 %)	<0.0001
Medium	20,795 (27.6 %)	<0.0001
	Large	41,260 (54.7 %)	<0.0001
Disposition		
Discharge to home	16,945 (22.5 %)	<0.0001
Home health care	15,795 (21 .0 %)	<0.0001
Against medical advice (AMA)	600 ( 0.8 %)	<0.0001

In-hospital mortality was 11.07%, the average length of stay was 13.8 days, and the hospital cost was 41,232.6 USD (Table 3).

**Table 3:** Primary outcome measures: in-hospital mortality, length of stay, and hospital cost

Outcomes	Results
In-hospital mortality	11.07 (p <0.0001)
Average length of stay	13.8 ( p <0.0001)
Hospital cost	41,232.6 USD (p<0.0001)

In this cohort of patients with enterococcal bacteremia, 25.91% (19,544) were suffering from congestive heart failure, 8.08% (6,110) from valvular disease, 11.87% (8,976) from neurological complications, 18.9% (14,256) from diabetes mellitus with complications, 28.52% (21,497) from renal failure, and 11.61% (8,749) of patients had obesity (Table 4).

**Table 4:** Enterococcal bacteremia and patient comorbidity CHF: congestive heart failure; DM: diabetes mellitus

Comorbidities	% of patients (Number of patients)
CHF	25.9 (19,544 ) p<0.0001
Valvular heart disease	8.1 (6,110) p<0.0001
Neurological disease	11.9 (8,976) p<0.0001
DM with complications	18.9 (14,256) p<0.0001
Renal Failure	28.5 (21,497 )< p<0.0001
Obesity	11.6 (8,749) p<0.0001

## Discussion

Enterococcal bacteremia has been significant in surgical ICUs and inpatients for a long time, but its significance is unclear in medical inpatients. Most of the studies done in the United States have used ICD-9 CM codes while looking for the outcomes among patients with enterococcal bacteremia. The ICD-10 CM has expanded to 19 times as many procedure codes as the ICD-9 CM and five times more diagnosis codes, and we used it for this study. Recent studies have shown *Enterococcus *as a significant pathogen in people with chronic illnesses. The emergence of *Enterococcus* and vancomycin-resistant Enterococcus (VRE) as significant hospital-acquired infections requires us to reassess nosocomial pathogens' epidemiology. Prior use of carbapenems and cefepime has been associated with an increased risk of acquiring enterococcal bacteremia in the first 48 hours in the ICU [4]. Previous antibiotic use has also been associated with antibiotic resistance among *Enterococcus*. A literature review has identified risk factors for death among patients with enterococcal bacteremia as surgery, nasogastric tube, arterial lines, and higher APACHE (acute physiological assessment and chronic health evaluation) score (Appendix 1), renal replacement therapy, cirrhosis, malignancy, and immunosuppression [5,6,7]. Many of these appear to be markers of the severity of the primary illness. Thus, the exact contribution of *Enterococcus* to mortality is difficult to ascertain.

Enterococcal bacteremia is associated with a higher prevalence of enterococcal endocarditis [3,8]. The presence of a prosthetic heart valve, community acquisition, three or more positive blood cultures, an unknown portal of entry, monomicrobial bacteremia, and immunosuppression are risk factors associated with a higher prevalence of endocarditis in enterococcal bacteremia [8]. *Enterococcus* accounts for 7.4% of all healthcare-associated infections [9]. The presence of VRE is associated with even higher healthcare costs. Our study aims to discuss the outcomes and associated comorbidities with enterococcal bacteriaemia using ICD-10 CM codes from the NIS database. In our research, the most prevalent comorbidity associated with enterococcal bacteremia was renal failure, followed by congestive heart failure, diabetes mellitus with complications, and neurovascular events like stroke. The high incidence of enterococcal bacteremia in renal failure can be attributed to increased use of urinary catheters, renal replacement therapies like dialysis, transplantation, and immunosuppression. Uremia is associated with immune failure due to uremic intoxication, altered renal metabolism of immunologically active proteins, T-cell dysfunction, and decreased antibody production in renal failure. In our study, CHF and valvular heart diseases are other significant comorbidity associated with enterococcal bacteremia. CHF is associated with increased use of pacemakers, implantable cardioverter defibrillators, and ventricular assist devices. These devices and valvular lesions provide *Enterococcus* a surface to adhere to and colonize. These are associated with increased enterococcal colonization and bacteremia. Angiotensin convertase enzyme (ACE) inhibitors are widely used in CHF. ACE is essential in the immune response of neutrophils, and ACE inhibitors cause a decrease in immune function [10].

A systemic review and meta-analysis by the WHO (European region) pooled all-cause mortality of hospital-acquired infections caused by *Enterococcus*, ranging between 14.3% and 32.3% (pooled estimate: 21.9%; 95%CI: 15.7-28.9, five studies) [11]. This rate is considerably higher than in our study, which is 11.07%. Unlike our study, which has data from NIS and includes a wide variety of patients from different settings, most of the studies included in the WHO (European region) meta-analysis were conducted in academic centers and tertiary care hospitals and involved mostly ICU, surgical wards, and burn unit patients. Thus, the patient representation was highly selective and derived from places where we have the sickest patients with high mortality and a chance of getting infections. Besides this, we have seen a decrease in the prevalence of VRE and *Enterococcus* in the United States in recent years [12] due to broader screening practices and implementation of contact precautions to control methicillin-resistant* Staphylococcus aureus *(MRSA) and VRE*.* This has helped in preventing nosocomial infections in people with multiple comorbidities. This may also affect the mortality in our study as compared to other studies. Enterococcal bacteremia is associated with an average stay of 13.8 days and an economic burden of 41,232.6 USD. Age-adjusted mortality among patients with enterococcal sepsis is 3.17 (3.09-3.25). Thus, enterococcal bacteremia cost both in terms of human resources and finances. The results of our studies are similar to the study done in Spain by Caballero-Granado et al. [13]; the effect of enterococcal bacteremia on mortality in this study was also attributable to the comorbidities. There was an increased length of stay in the hospital, along with increased healthcare costs. According to this study, the mortality rate attributable to enterococcal bacteremia was not significant. However, a stratified analysis of the same data shows that the attributable mortality rate was significant if inappropriate antimicrobials were used or if the patients developed a hemodynamic compromise.

We conducted a retrospective epidemiological study using data from a large United States inpatient population pool. The size of our patient population is its biggest statistical strength as we analyzed patient demographics, healthcare specifics like insurance, and hospital type, and then analyzed mortality rate, length of hospitalization, and healthcare cost. It is also a strength that patients were not selected from any specific group of inpatients, and it included smaller community centers as well as larger academic tertiary care centers; thus, these results can be generalized and used in planning further prospective studies that are stratified to find the morbidity and mortality risk attributable to *Enterococcus* in hospitalized patients and help in policy planning and forming appropriate prevention and treatment guidelines considering the risk and cost-benefit analysis. A significant limitation of this study is that retrospective studies have missed data, reducing the study's power. Our study is based on data derived from ICD codes. A study design based on data derived from patients' charts will be more informative. 

## Conclusions

Enterococcal bacteremia is a significant nosocomial infection with a preference for the elderly. It is associated with increased mortality; however, with our study design, it is hard to comment if the increased mortality is attributable to comorbidities or the infection itself. However, morbidity is represented by a more extended hospital stay and significantly high healthcare expenditure compared to average healthcare costs. The most important comorbidities associated with enterococcal bacteremia are renal failure, cardiac failure, cardiac valvular diseases, stroke, diabetes with complications, and obesity. Implementing contact precautions to contain the spread of MRSAand VRE has helped bring down nosocomial infections. Hand hygiene is a simple but effective method to control the spread of diseases. However, VRE continues to be a significant clinical and epidemiological problem. We can plan prospective studies based on these results to quantify the effect of VREon mortality, morbidity, and burden on healthcare resources.

## Appendices

Acute physiological assessment and chronic health evaluation (APACHE) score

APACHE score [14] is an illness severity score commonly used in critical care medicine to predict mortality upon admission to an intensive care unit. 

APACHE score = Acute Physiology Score (APS) + Age Points + Chronic Health Points

**Table 5:** Acute physiologic score (APS) Fio2: fraction of inspired oxygen; PaO2: arterial oxygen pressure

Criteria	Points
Physiology score (APS)	Sum of the 12 individual variable points
Temperature (rectal)	
Mean arterial pressure	
Blood pH	
Heart rate	
Respiratory rate	
Sodium	
Potassium	
Creatinine	
Hematocrit	
While blood cell count	
Glasgow coma scale	
Blood oxygenation A-a gradient (if Fio2 is more than or equal to .5) PaO2 (if FiO2 is less than 0.5)	

**Table 6:** Other criteria used in APACHE Score APACHE: acute physiological assessment and chronic health evaluation

2. Age points	
3. Chronic health conditions like organ insufficiency or immunocompromised state	
